# Modeling the transmission of community-associated methicillin-resistant *Staphylococcus aureus:* a dynamic agent-based simulation

**DOI:** 10.1186/1479-5876-12-124

**Published:** 2014-05-12

**Authors:** Charles M Macal, Michael J North, Nicholson Collier, Vanja M Dukic, Duane T Wegener, Michael Z David, Robert S Daum, Philip Schumm, James A Evans, Jocelyn R Wilder, Loren G Miller, Samantha J Eells, Diane S Lauderdale

**Affiliations:** 1 Decision and Information Sciences Division, Argonne National Laboratory, 9700 S. Cass Ave., Bldg 221, Argonne, IL 60439, USA; 2 Computation Institute, University of Chicago, Chicago, IL 60637, USA; 3 Applied Mathematics, University of Colorado Boulder, Boulder, CO 80309, USA; 4 Psychology, Ohio State University, Columbus, OH 43210, USA; 5 Department of Medicine, University of Chicago, Chicago, IL 60637, USA; 6 Health Studies, University of Chicago, Chicago, IL 60637, USA; 7 Pediatrics, University of Chicago, Chicago, IL 60637, USA; 8 Sociology, University of Chicago, Chicago, IL 60637, USA; 9 Harbor-UCLA Medical Center, Division of Infectious Diseases, Torrance, CA 90509, USA

**Keywords:** MRSA, Agent-based model, Infectious disease model

## Abstract

**Background:**

Methicillin-resistant *Staphylococcus aureus* (MRSA) has been a deadly pathogen in healthcare settings since the 1960s, but MRSA epidemiology changed since 1990 with new genetically distinct strain types circulating among previously healthy people outside healthcare settings. Community-associated (CA) MRSA strains primarily cause skin and soft tissue infections, but may also cause life-threatening invasive infections. First seen in Australia and the U.S., it is a growing problem around the world. The U.S. has had the most widespread CA-MRSA epidemic, with strain type USA300 causing the great majority of infections. Individuals with either asymptomatic colonization or infection may transmit CA-MRSA to others, largely by skin-to-skin contact. Control measures have focused on hospital transmission. Limited public health education has focused on care for skin infections.

**Methods:**

We developed a fine-grained agent-based model for Chicago to identify where to target interventions to reduce CA-MRSA transmission. An agent-based model allows us to represent heterogeneity in population behavior, locations and contact patterns that are highly relevant for CA-MRSA transmission and control. Drawing on nationally representative survey data, the model represents variation in sociodemographics, locations, behaviors, and physical contact patterns. Transmission probabilities are based on a comprehensive literature review.

**Results:**

Over multiple 10-year runs with one-hour ticks, our model generates temporal and geographic trends in CA-MRSA incidence similar to Chicago from 2001 to 2010. On average, a majority of transmission events occurred in households, and colonized rather than infected agents were the source of the great majority (over 95%) of transmission events. The key findings are that infected people are not the primary source of spread. Rather, the far greater number of colonized individuals must be targeted to reduce transmission.

**Conclusions:**

Our findings suggest that current paradigms in MRSA control in the United States cannot be very effective in reducing the incidence of CA-MRSA infections. Furthermore, the control measures that have focused on hospitals are unlikely to have much population-wide impact on CA-MRSA rates. New strategies need to be developed, as the incidence of CA-MRSA is likely to continue to grow around the world.

## Introduction

*Staphylococcus aureus* is a common cause of human bacterial infections. It is generally a commensal organism, and it is estimated that 25-40% of the population are colonized in the nasopharynx at any given time. While colonization is asymptomatic, colonized individuals may develop an active infection [1]. Colonization may be of short or long duration, and often clears without causing an infection. Transmission is believed to be largely via skin-to-skin contact with either a colonized or infected individual. New *S. aureus* isolates resistant to β-lactam antibiotics were identified in the 1960s among hospitalized patients [2,3]. Called methicillin-resistant *Staphylococcus aureus* (MRSA), these isolates rapidly became an important cause of nosocomial infections, particularly among patients with procedures or devices that pierce the skin. These are referred to as “health care-associated” MRSA (HA-MRSA) strains [4].

MRSA epidemiology changed in the 1990s in many countries when new MRSA strain types that were genetically distinct from HA-MRSA were diagnosed among individuals in the community who did not have healthcare exposures. The transmissibility and types of HA-MRSA and community-associated MRSA (CA-MRSA) infection differ [5]. The problem of CA-MRSA has been particularly severe in the United States, where incidence increased exponentially in the early 2000s [6]. In the United States, early reports of CA-MRSA infections came from inner city populations [7], and outbreaks were reported among members of contact sports teams, jail detainees, and army personnel, all in places where people interact in close quarters [8]. By 2004, one of the CA-MRSA strain types, USA300, became the most frequent cause of skin and soft tissue infections seen in U.S. emergency departments [9]. While uncomplicated skin infections are the most common manifestation, CA-MRSA infections may be invasive and even fatal.

Public concern about MRSA increased when the Centers for Disease Control and Prevention (CDC) estimated that in 2005 there were more deaths from invasive MRSA infections than from HIV-AIDS in the U.S. [10]. While infections from HA-MRSA strains rarely occur outside of the healthcare setting, infections caused by CA-MRSA strain types have become common in hospitals in the U.S. [8]. Invasive MRSA deaths are caused by both HA-MRSA and CA-MRSA strain types. Since 2001, several states enacted laws designed to slow the dissemination of MRSA, all focused on healthcare settings (Committee to Reduce Infection Deaths. State Legislation & Initiatives on Healthcare-Associated Infections. http://www.hospitalinfection.org/legislation.shtml). The other public health response in the U.S. has been educational, such as public service announcements to raise public awareness of MRSA infections and to encourage the use of preventive measures such as the covering of skin lesions to reduce transmission (STOP MRSA Now. http://www.stopmrsanow.org/public-service-announcement.html). There are no major public or private efforts aimed at controlling the spread of MRSA colonization in the community from person to person in the absence of infection.

Epidemiologic studies can be used to estimate the incidence and risk factors for CA-MRSA infections, or the prevalence of colonization. However, it is difficult to perform a study that examines the actual transmission dynamics of asymptomatic MRSA colonization in any population.

This paper describes the structure and results of a new, fine-grained agent-based model of CA-MRSA transmission dynamics and infection in Chicago aimed at augmenting the value of existing epidemiological data and evaluating current public policies related to MRSA. Computational models have become valuable tools in understanding infectious disease dynamics, including assessments of the potential impact of public health interventions such as isolation, school closures or vaccines. While there have been small-scale models of HA-MRSA within hospitals and other resident healthcare facilities [11-18] and examinations of multiple interacting hospitals [19-21], attempts to model MRSA in the community have been limited [22,23]. Our model represents the spread of CA-MRSA in the population of Chicago, for which we have estimates of temporal and geographic trends based on clinical data from 2001 to 2010.

We used the model to determine the types of places in Chicago most important for the transmission of CA-MRSA, examining places such as households, the County jail, hospitals, and schools, and the relative contributions of the colonized and infected states to transmission.

## Methods

**Figure 1:**
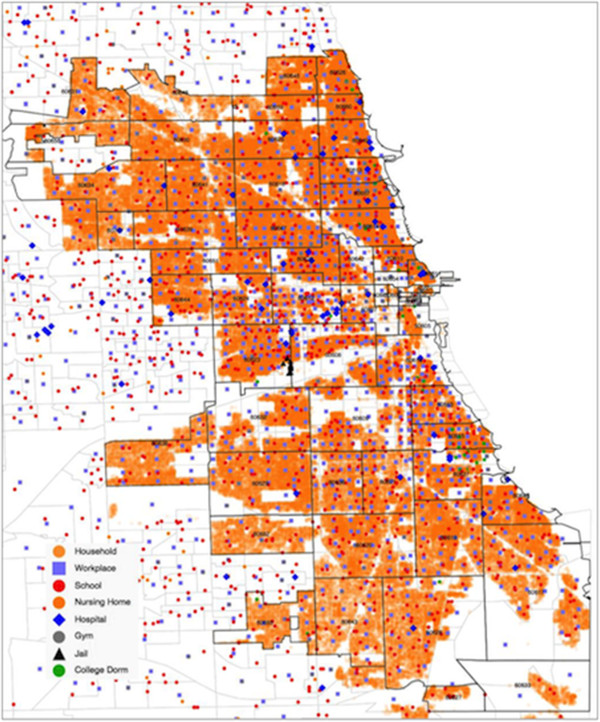
Places included in Chicago MRSA agent-based model.

The baseline data for the model is a synthetic population developed by Wheaton and others [24] derived from combined U.S. Census files. The sociodemographic attributes of the synthetic population match that of the enumerated population in the 2000 Census for Chicago. Each agent resides in a household and has sociodemographic characteristics (e.g., race/ethnicity, age, gender, educational attainment, income). Every place in the model, including households, schools, hospitals and workplaces, has geographic coordinates (Figure 1). About three million individual agents move to and from 1.2 million places on an hourly basis over a period of up to 10 years. Places are categorized as having different levels of physical contact, because MRSA is transmitted primarily by direct skin-to-skin contact. The risk of transmission to or from an agent depends on the disease status (uncolonized, colonized or infected) of other agents co-located at the same time in the same place and on the overall level of physical contact that occurs in that type of place.

### Agent activities and contacts

CA-MRSA presents challenges for computational epidemiologic modeling that differ from more commonly modeled diseases that spread in the community, such as influenza, because MRSA has a colonized state. Modeling CA-MRSA also means representing individuals’ behaviors related to disease transmission and response to infection. The kinds of places that need to be represented in the model also differ from other diseases. Early outbreaks in urban jails pointed toward jails [25-27] as potentially playing a key role in transmission, necessitating their inclusion in a population-based model.

We combine several publicly available national data sources to model activities of the synthetic agent population. Each agent has a daily activity profile that determines what times throughout the day he or she occupies each location. Social contact between agents occurs when multiple agents occupy the same location at the same time. The synthetic population assigns agents to households, workplaces and schools (for those of school age) [24]. Because daycare centers are not represented in the synthetic population, we generated pseudo-daycare centers at the same geographical locations as the schools in the model and assigned each agent attending daycare to the closest one. Since daycare children do not collocate in the same classrooms as the school children, the exact locations of the daycare centers do not affect the final results, as long as they are in the neighborhoods of the children who attend. Each household is randomly assigned three other households for potential visits from the same census block group and one household from elsewhere in the city, and one of these four households is randomly selected whenever an agent has an activity in another household (e.g., babysitting or socializing). Hospitals and gyms are in the synthetic population because they are workplaces, but we assign agents to them for athletic activities and hospitalizations, using the geographically closest one. Finally, the model includes a jail corresponding to the Cook County Jail (one of the largest jails in the U. S.).

Activity profiles are empirically based on 24-hour time diaries collected as part of the U.S. Bureau of Labor Statistics’ annual American Time Use Survey (ATUS) for individuals aged 15 years and older and from the Panel Study of Income Dynamics (PSID) for children younger than 15 years. Both are nationally representative samples and collected diary data on randomly assigned days. The diary records each activity during the 24-hour period (start/stop times, location and others present). We simplify the data, focusing on features of greatest relevance for MRSA transmission. Each place/activity is categorized according to (1) the likelihood of physical contact that would increase transmission probability, and (2) the likelihood of injuries, cuts and abrasions that would increase the probability of transition from the colonized to the infected state. Athletic activities, for example, are considered high risk for both (1) and (2), regardless of where they occur.

Two profiles (one weekday and one weekend) from respondents living in metropolitan areas are assigned to each agent in the model. This is done by randomly matching each agent with an ATUS or PSID respondent who is either identical or similar with respect to sociodemographic characteristics. While these profiles are currently used for the entire model run, it would be straightforward to incorporate variation in activity profiles or other within-agent changes in profiles that respond to disease or other events.

Days spent in hospitals or jail are both relevant for MRSA transmission but are not included in the time diaries, which exclude institutionalized individuals. To assign hospital stays to agents, we use data on hospital nights per year from the 2010 National Health Interview Survey (NHIS), matched to agents by sociodemographics. Five matches are made per agent to be used in successive years, and these are repeated for ten-year model runs. Hospitalization timing is random throughout the year. For risk of being detained in jail, we generate a yearly probability for each agent so that the total number of admissions and their distribution by gender, age, race and zip code matches data from the 2006 National Jail Survey (for Chicago) and the Arrestee Drug Abuse Monitoring (ADAM) program as closely as possible. Agents are placed in jail up to once per year according to this probability, with a length of stay drawn randomly from the actual distribution at the Cook County Jail.

**Figure 2:**
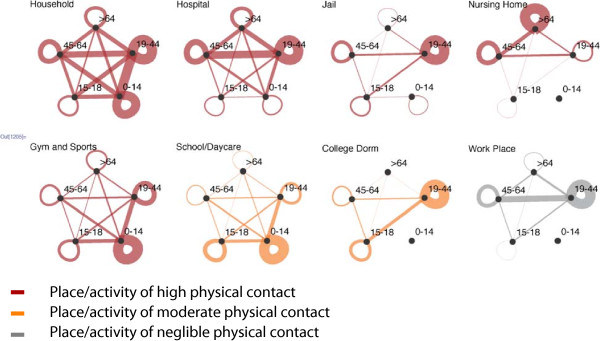
**Age group contacts by place/activity.** The width of the lines connecting the nodes represents the relative number of contacts between individuals in each age group. The width of the loops similarly indicates the relative numbers of contacts with the people within the same age group.

Rather than using a mathematically generated contact network, the contact network is an emergent characteristic of this model, driven by geography and co-participation in activities [28]. Within each physical location, random mixing is assumed to occur among all present with one exception. Within schools and daycare centers, children are assigned to compartments of size 30 as a proxy for grade or group, so that their contacts are limited to other children in the same classroom. Each hour, four children in the same compartment are randomly selected for direct contact risk. Similarly, individuals detained in the jail are placed in compartments of 30 people. Figure 2 shows the extent to which individuals in different age groups have contact with individuals by age group in key types of locations. Each hour, individuals in the same location are in contact with each other, such that two individuals in the same house for three hours are considered three contact events for each of them. Overall, in a ten-year model run there were 4.125 × 10^12^ contact events, an average of 39 contact events per day per agent.

### Disease states and transitions

**Figure 3:**
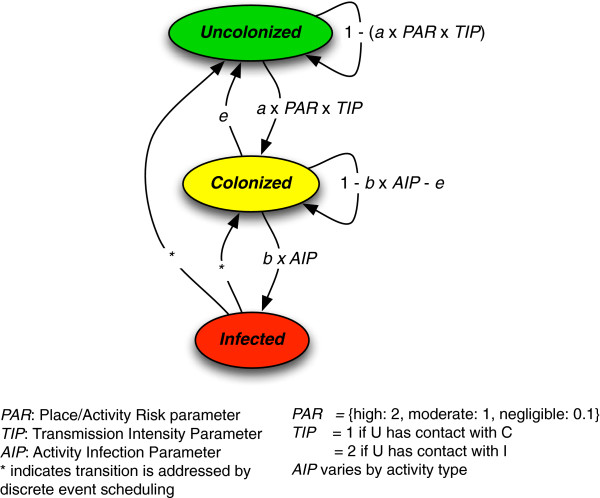
Disease state transitions in MRSA agent-based model.

Each agent in the model is in one of three disease states at any time: colonized (denoted by C), uncolonized (denoted by U), or infected (denoted by I). Individuals transition between these states, and individuals return to being susceptible after an infection or colonization, consistent with the observed frequency of repeat infections [29]. Figure 3 summarizes the disease states and transitions. A probabilistic discrete-time state transition approach is used to model change in agent disease states, combined with a discrete event scheduling approach with estimated distributions for the duration of time spent in the states involved. Estimated transition probabilities are used for the transitions U → C, C → U and C → I, and event scheduling determines transitions for I → C and I → U. We assume perfect social mixing among individuals within a given location, except for schools and jails (as described above).

Transition from Uncolonized to Colonized State. An uncolonized individual may become colonized upon contact with a colonized or infected individual. We define the parameter *a* as the estimated transition probability of an uncolonized individual (in disease state U) becoming colonized (transitioning to disease state C) due to contact for one hour with a colonized individual. Parameter *a* is the only disease state transition parameter in the model that is the result of physical contact between individuals. To consider a higher probability of becoming colonized upon contact with an infected individual, we define the Transmission Intensity Parameter (TIP), which scales the transmission rate. In the base case, we leave this parameter with a value of 1, but we investigate the sensitivity of our findings with respect to this parameter. For sensitivity analyses, the value of TIP, based on expert judgment of infectious disease physicians (MZD and RSD), is set as twice the transmission risk per hour when there is contact with an infected rather than a colonized agent.

The likelihood of transmission also depends on the amount of physical contact in the place/activity: high, moderate or negligible. Place/activities are assigned one of three levels of risk (Figure 3). This scaling factor is denoted as the Place/Activity Risk parameter (PAR). Equation 1 summarizes the state transition process from the uncolonized to the colonized state.

(1) $$\mathrm{Pr}{\left[U\to C\right]}_{s}={\mathrm{TIP}}_{s}\times \mathrm{PAR}\times a$$

where Pr[U → C]_*s*_ is probability that an uncolonized individual becomes colonized through contact with an individual in state *s,* of C or I, and TIP_*s*_ depends on the state of the contacting individual. MRSA risk categories by place and activity are summarized in the technical appendix (Additional file 1: Table S1).

Transition from Colonized to Uncolonized State. An individual may spontaneously become uncolonized, as the individual’s defenses eliminate the bacteria, or may remain colonized over an extended period. The hourly probability of a transition is denoted by parameter *e*, estimated as described below:

(2) $$\mathrm{Pr}\left[C\to U\right]=e$$

Transition from Colonized to Infected State. Colonized individuals may develop infections. This may be a result of a skin abrasion or other injury, or of an indeterminate process. The relevant probability, represented as parameter *b*, is estimated as described below. In the model,

(3) $$\mathrm{Pr}\left[C\to I\right]=\mathrm{AIP}\times b$$

where *b* is the probability of a colonized individual becoming infected per hour, and AIP (Activity Infection Parameter) is a parameter that reflects the relative likelihood that a place/activity results in an infection. Activities with a higher risk of the skin being abraded or punctured (such as may happen at a sports activity or in a hospital, jail or daycare center) have elevated risk of infection. We assume that an individual always passes through the colonized (C) state prior to becoming infected (I), although this is a minor assumption as the duration of colonization may be as brief as one hour.

Transition from Infected to Colonized and Uncolonized States. Infections vary in length, averaging about two weeks, based on clinical observations and expert judgment. An infected individual may seek treatment or care for the infection without seeking professional medical care. We collected national survey data through the Time-sharing Experiments for the Social Sciences (TESS) to determine individuals actual and hypothetical care-seeking behavior in response to MRSA-like skin infections. Individuals consistently reported that they had or would seek clinical care about half the time (Wilder JR, Wegener D, David MZ, Macal CM, Daum R, Lauderdale DS: *A national survey of skin infections, care behaviors and MRSA knowledge*, submitted). Almost all MRSA skin infections would resolve whether or not care is sought, although clinical attention likely decreases the duration of infection. When the infection is resolved, the relative likelihood of ceasing to be colonized with MRSA may be higher when the infection is treated with antibiotics by a clinician. Both because clinicians may treat infections with antibiotics and because they would give instructions on how to care for an infection, the proportion uncolonized after infection is greater for those who seek professional medical care than those who do not. These transitions and contingencies are graphically represented in the behavioral model presented in Additional file 1: Figure S2 in the technical appendix.

### Estimating disease transmission and transition parameters

We used published data for initial estimates of colonization rates [30], decolonization rates [31], and infection rates [32]. We derived household transmission rates from data described in Miller et al [30]. and used bootstrap resampling methods to calculate confidence intervals, which we used as plausible ranges for transmission rates in the simulation. Disease state transition parameters are estimated using a discrete-time Markov chain approach [33].

We then ran the model over the space of plausible transmission parameters. In doing so we found that some combinations of transmission parameters produced results that closely matched the CA-MRSA build-up meta-analysis estimates [6] that occurred in the period 2001 – 2010 in Chicago, while others did not. The identification of transmission parameters is more fully described in the technical appendix (Additional file 1: Figure S3). The agent disease state transmission/transition parameters are shown in Table 1.

**Table 1:** Agent disease state transmission/transition parameters

**Parameter (Probability/Hour)**	**Range of values tested**	**Range for good model fits**	**Best fit of disease state transition probabilities**
*a*	2.053 × 10^−5^ - 6.160 × 10^−5^	3.080 × 10^−5^ - 5.134 × 10^−5^	4.107 × 10^−5^
*b*	2.625 × 10^−6^ - 7.875 × 10^−6^	2.625 × 10^−6^ - 3.938 × 10^−6^	3.938 × 10^−6^
*e*	7.961 × 10^−5^ - 2.388 × 10^−4^	7.961 × 10^−5^ - 1.194 × 10^−4^	1.194 × 10^−4^

Notes: *a* is the probability per hour of an uncolonized agent who is located in a place with one or more colonized individuals transitioning to colonized state. *b* is the probability per hour of a colonized agent spontaneously transitioning to infected state. *e* is the probability per hour of a colonized agent spontaneously transitioning to uncolonized state.

### Initial conditions

All runs were initialized with the number of infected individuals at 104 and the number of colonized individuals at 21,944 for the base year, 2001. The initial number of infected is consistent with observed data [17]. The number of colonized individuals is not observed, but is based on national colonization data in the same year that was collected as part of the National Health and Nutrition Examination Survey, a nationally representative survey collected by the National Center for Health Statistics. From 2001 through 2004, they included a special assessment of nasal colonization for MRSA [34]. We adjusted the colonization rate they found to account for an underestimate because only one body site (nares) was checked for colonization. Infected and colonized individuals were randomly located in the zip codes that had MRSA cases in 2001, drawing on data described in Hota et al. [35].

### Modeling behaviors

We developed a flexible framework for modeling behavior of individuals in response to infection, which could be expanded to include the behavior of healthcare workers. Based on available evidence [30,31] and expert opinion, clinical care may reduce the duration of infection and decrease the probability of being colonized after the infection resolves. Thus, we permit infected individuals to vary in the likelihood that they seek medical care, with overall probability estimated from our TESS survey. The behavioral model is presented in the technical appendix (Additional file 1: Figure S2).

### Model validation

There are generally accepted processes for validating large-scale simulation models [36]. Validating a social systems model that contains elements of human behavior to the point of numerical equivalence between model outputs and observed phenomena is usually not possible and is generally not considered an attainable goal [37]. Rather, a series of validation tests are performed to establish credibility for the model for its intended use. Approaches to validation include establishing face validity (domain experts’ judgmental assessments of model mechanisms and results), stochastic equivalence (adherence of the distribution of model outputs to estimated uncertainty ranges for observed data), alternative history explorations (ability of the model to produce alternate histories), model falsification (parameter sweeps over a range of plausible assumptions designed to reveal implausible cases), and model docking (comparing results of different types of models of the same system). Each of these approaches was used in the iterative development of the CA-MRSA ABM. Tests that support the validity of the model include recorded infection incidence by zip code [35], jail stay annual data (various sources), hospital visits annual data (various sources), and colonization data from diverse studies.

### Platform

The ABM is implemented in Repast, a general-purpose agent-based modeling toolkit. The Repast suite includes the Java-based Repast Simphony [38] and the C++ − based Repast for High Performance Computing (HPC) [39]. The initial CA-MRSA ABM prototype was implemented in Repast Simphony for a small region of three Chicago zip codes [40]. The prototype was translated into Repast HPC for large-scale model runs. The CA-MRSA ABM is designed to run on computing platforms that include desktop computers, computing clusters (e.g., the Blues and Fusion clusters at Argonne National Laboratory), and specialized high performance computers (e.g., the IBM Blue Gene class of machines at Argonne National Laboratory).

### Stochastic variability

The CA-MRSA ABM is inherently a stochastic model, with random variation affecting both disease transmission (uncolonized to colonized transition) and transition (the length of time in the colonized or infected states). To properly characterize uncertainty requires that, for each case run with the model, multiple simulations be run in which the initializing random number seeds are varied. Studies are described in the technical appendix of the appropriate number of simulation runs to characterize uncertainty of the model outputs (Additional file 1: Figure S5). The results show that the moving average of the estimated variance decreases continuously from 15 to about 28 runs and then stabilizes, indicating that batch sizes of a minimum of 28 runs are adequate to estimate of model output statistics. Results presented here are based on 32 runs, which was found to adequately characterize the inherent uncertainty in the model.

## Results

**Figure 4:**
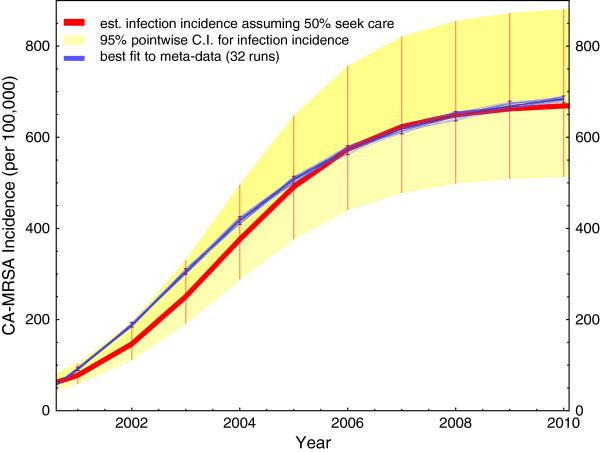
Comparison of best fit from simulations to estimated CA-MRSA infection incidence.

Figure 4 shows the meta-analysis estimate of the actual CA-MRSA trend in Chicago from 2001 to 2010 in red [6], which has been adjusted for an underestimate because the incidence data derive from clinical records only includes MRSA infected patients who seek medical care. The yellow band is a point-wise 95% C.I. representing uncertainty around the Chicago incidence estimate. The blue band corresponds to the results from 32 model runs using the best fitting transmission parameters, and its width indicates variability over the runs. The narrowness of this blue interval demonstrates that, at least point-wise, there is little stochastic variability in the results of the model (conditional on these values of the parameters), and the vast majority of the variation in Figure 4 is due to intentional variation in the values of the model parameters.

**Figure 5:**
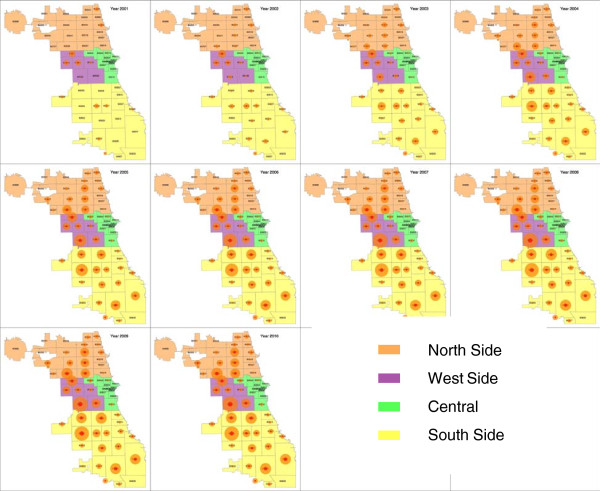
**Model results of geographic distribution of CA-MRSA colonizations and infections, by zip code and year.** Each circle shows the relative number of cases of CA-MRSA colonizations (outer circle) and CA-MRSA infections (inner circle).

Over the same years, cases were observed to spread from the initial concentration in inner city neighborhoods to a more diffuse pattern across the city. Figure 5 shows the model output by zip code and year, with the diameter of the circles indicating the numbers of newly colonized agents (larger orange circles) and incident infections (smaller red circles) in each zip code by year. During the first five years, there is a spread from a small number of zip codes to a citywide problem, and the pattern then remains similar for the last five years.

Over each ten-year run, there were about 2.1 × 10^6^ new colonization events, or 72 colonization events per 1000 person-years, and there were about 116,000 new infections, or about 4 per 1000 person-years. Among the 2.1 × 10^6^ new colonizations, 98.6 percent were the result of contact with a colonized individual and 1.4 percent were the result of contact with an infected individual.

By far the most common geographic location for colonization transmission events was households (including both an individual’s own household and ones they visited), accounting for 65.1 percent of newly colonized agents. Schools or daycare centers were the next most frequent, accounting for 15.8 percent. Hospitals accounted for only 7.8 percent of newly colonized agents. Initial reports of CA-MRSA outbreaks in the early 2000s came from jails and sports teams. However, jails and athletic activities were less frequent locations for new colonizations, accounting for 2.9 percent and 5.6 percent, respectively (Table 2).

**Table 2:** Model results on distribution of new colonizations by disease state of contact

**Place/Activity**	**Colonization incidence (per 1000 person-years)**	
	**Due to contact with colonized individual**	**Due to contact with infected individual**
Household	49.9	0.976
School/Daycare	12.3	0.072
Hospital	6.1	0.024
Athletic activity	4.4	0.016
Jail	2.3	0.015
College dorm	1.1	0.010
Nursing home	1.0	0.009
Workplace	0.1	0.007

### Sensitivity analyses

A set of experiments was designed to test the robustness and sensitivity of the model results to three types of parameters whose initial values were set based on expert judgment. (1) Different types of places provide opportunities for lesser and greater levels of physical contact, and the Base Case values reflected those differences. PAR indicates the relative likelihood of physical contact between individuals at various places, with PAR >1 indicating relatively more contact. For sensitivity analysis, the value of PAR did not vary by place. (2) Individuals with active infections may be more likely to spread colonization than colonized individuals. TIP allows infected individuals to be more likely (TIP > 1) to spread colonization than colonized individuals. Sensitivity analyses set TIP at 2. (3) Physical activities (including sports activities) provide more opportunities for an individual to transition from the colonized to infected states because of increased likelihood of cuts and bruises. The parameter AIP is applied to the transition parameter *e* to indicate activities that are more likely (AIP > 1) to result in self-infection. Sensitivity analyses did not vary AIP by place. In summary, we carried out sensitivity analyses to test the effects of varying the values of PAR, TIP and AIP.

Table 3 shows the values of PAR, TIP and AIP for sensitivity analyses. Table 4 shows the results of the sensitivity analyses. There is little variation in the proportion of all disease transition events that are colonization events across all cases (column 2). Compared the Base Case, the AIP Sensitivity Case shows the greatest change, an increase from 94.6% to 96.9%. Such an increase is not surprising for this case as it reduces the probability of individuals transitioning from the colonized to infected states in physically active places.

**Table 3:** Set up for sensitivity analyses of transmission parameters

**MRSA Risk Category**	**PAR base case value**	**PAR sensitivity case**	**TIP sensitivity case**	**AIP sensitivity case**
Households	2	1	2	1
Hospitals	2	1	2	1
Jails	2	1	2	1
Nursing homes	2	1	2	1
Gyms	2	1	2	1
Schools/Daycare	1	1	2	1
College dormitories	1	1	2	1
Workplaces	0.1	0.1	2	1

Notes: AIP values are assigned according to the specific activity that an individual is engaged in at a specific place and time during the simulation. TIP Base Case values are all 1. AIP Base Case values are 1 (non-physical activities) or 2 (physical activities).

**Table 4:** Sensitivity analyses results

**Case**	**% Disease transition events that are colonizations**^**1**^	**% Disease transition events that occur in households**^**2**^
Base case	94.6	72.9
PAR sensitivity	94.7	53.9
TIP sensitivity	94.5	82.2
AIP sensitivity	96.9	72.7

Notes: A disease event transition is defined here as a transition from the uncolonized state to the colonized state or a transition from the colonized state to the infected state. 1: Computed as (Number of Colonizations)/(Number of Colonizations + Number of Infections). 2: Computed as (Number of Colonizations and Infections That Occur in Households)/(Number of Colonizations + Number of Infections).

There is greater variation in the portion of colonization events that occurs in households (column 3) across all sensitivity analyses. The largest contrast to the Base Case is when the PAR is set to 1 in all places. Then the proportion of new colonizations that take place in households is greatly reduced, although still a majority (53.9% compared to 72.9%). However, the assumption that all places are equally likely to provide opportunity for skin-to-skin contact, which is generally thought to be necessary for transmission, is not plausible.

## Discussion

Using a novel fine-grained agent-based model of the population of Chicago, we found that the great majority of new CA-MRSA colonization events occurred in households, where most individuals spend substantial time and have a relatively high level of physical contact with others. Schools and daycare centers played the largest role in spreading colonization among households. Although outbreaks in jails and sports teams were prominently featured in early reports about CA-MRSA, over the course of the ten-year runs of the model, they are responsible for relatively low percentages of new colonization events. Nonetheless, athletic activities of all kinds did account for more than five percent of colonization events and could have a high attributable risk for individuals who engage in athletic activities. During the first five years (2001 – 2005) model results show spread from a small number of zip codes concentrated on the south and west sides of the city to citywide incidence, and that pattern remains similar for the next five years (2006 – 2010). These data are consistent with the findings of Hota et al. [35].

We also found that the overwhelming majority of new colonizations are the result of a contact with a colonized individual rather than an infected individual, even when we set the probability of transmission as twice as great when a susceptible individual spends time with an infected person, compared with a colonized person. However, since colonization is so much more common than infection and lasts much longer, an intervention which focuses solely on clinically apparent infections to reduce transmission is unlikely to have much impact on overall incidence. These results suggest that a particularly effective intervention might be one that prevented transmission from asymptomatic MRSA carriers within households to their household contacts. These findings challenge current public policy in the U.S., which focuses on the control of MRSA in the healthcare setting or among people with active infections.

Our model has several novel features that represent important aspects of the epidemiology of CA-MRSA and its transmission. Time is represented at the hourly level allowing us to incorporate detailed empirical time-use data into the model. Space is represented by geo-located place, and the contact network is an emergent characteristic of agents located in the same physical places. We have used the census sociodemographic characteristics of agents (i.e., age, sex, race/ethnicity, education, years in school, employment status and household income) to probabilistically link activity profiles and probabilities of hospitalization and jail detention to agents. In addition, the places/activities are graded with respect both to the level of direct physical contact likely to occur there, which affects the transition from the uncolonized to the colonized state (conditional on an individual with MRSA being in the same location), and to the likelihood of skin abrasions or punctures, which affects an individual’s transition from the colonized to the infected state. We have shown that the outputs of the model are similar to the temporal and geographic trends in CA-MRSA incidence in Chicago. This does not directly validate the transmission parameters or the contact network. However, the similarity of our model output to the descriptive epidemiology of CA-MRSA in Chicago will allow us to use the model to estimate the magnitude of likely effects both of public health and clinical interventions that have been implemented – generally without evidence – and of interventions that may be implemented in the future. Our findings suggest that novel public health approaches are needed to decrease the dissemination of CA-MRSA. The most effective interventions would prevent transmission from asymptomatically colonized people to their household contacts.

The most prominent type of public health intervention aimed at the control of MRSA transmissions to date has been the laws passed by some states that mandate screening and isolation procedures for patients entering hospitals. With only 7.8 percent of new colonizations estimated to occur in hospitals, this approach will have limited impact and concentrates resources on a type of location that seems to be responsible for less than one-tenth of transmissions.

Nevertheless the model has limitations. The results from any model are conditional on the assumptions made in deriving the model relations and on the data employed for that purpose. Some of these assumptions concern phenomena in part that are not directly observable, such as the number of people colonized with CA-MRSA. In the CA-MRSA ABM, the assumptions cover the three main model areas: contact, transmission, and behavior. We assume the activity profiles and time use surveys upon which the contact model is based, which are national in scope, are applicable to the Chicago area. We assume that assigning activity profiles based on demographic characteristics adequately captures the relevant heterogeneity in behavior, and that people continue in these activity patterns throughout the period covered by the simulation. We assume that the rules for matching people to associated hospitals, the jail, schools, and workplaces reflect a reasonable approximation to the real world. We assume that CA-MRSA transmission can be modeled accurately at the fine-grained compartment level based on agents’ behaviors and “micro-interactions,” such as skin contact based on co-location, and we assume random mixing among individuals at each location at the same time. These assumptions are explicit in the model, and can be easily modified as new data or alternate hypotheses become available and as the processes mentioned above are better understood. In the future, it will be possible to further justify these assumptions or modify them as necessary, thereby increasing the accuracy of the model and our confidence in its predictions as we experiment with novel interventions.

## Conclusions

In summary, our novel agent-based model of CA-MRSA in a large U.S. city demonstrated that current interventions aimed at controlling the spread of MRSA are unlikely to succeed in reducing overall CA-MRSA incidence. According to the results of our model, the most effective control strategy would be one that reduces CA-MRSA transmission within households, particularly from colonized to uncolonized contacts. Our findings suggest that current paradigms in MRSA control in the United States cannot be very effective in reducing the incidence of CA-MRSA infections. Furthermore, the control measures that have focused on hospitals are unlikely to have much population-wide impact on CA-MRSA rates. New strategies need to be developed, as the incidence of CA-MRSA is likely to continue to grow around the world.

### Supporting information

The Repast HPC computer code for the complete CA-MRSA ABM model is available at https://github.com/contact-contagion/mrsa-c.

## Competing interests

The authors declare that they have no competing interests.

## Authors’ contributions

CMM, DSL, MJN, RSD and MZD designed the research; MJN and NC implemented the model; CMM, DSL, MJN, NC, VMD, DTW, MZD, RSD, PS, JAE, JRW performed the research; LGM and SJE provided data for estimating model parameters; CMM, DSL and MZD analyzed the data; and CMM, DSL, RSD, MZD, and PS wrote the paper. All authors read and approved the final manuscript.

## Supplementary Material

Additional file 1 Technical appendix: supplemental information. Click here for file

## References

[B1] Lowy FD *Staphylococcus aureus* infections N Engl J Med 1998 339 8 520 532 10.1056/NEJM199808203390806 9709046

[B2] Jevons MP “Celbenin” - resistant Staphylococci Br Med J 1961 1 5219 124 125 PMC1952889 14447241

[B3] Barrett FF McGehee RF Finland MN Methicillin-Resistant *Staphylococcus aureus* at Boston City Hospital — Bacteriologic and Epidemiologic Observation N Engl J Med 1968 279 441 448 10.1056/NEJM196808292790901 4232865

[B4] Chambers HF Deleo FR Waves of resistance: *Staphylococcus aureus* in the antibiotic era Nat Rev Microbiol 2009 7 9 629 641 10.1038/nrmicro2200 19680247 PMC2871281

[B5] Naimi TS LeDell KH Como-Sabetti K Borchardt SM Boxrud DJ Etienne J Johnson SK Vandenesch F Fridkin S O'Boyle C Danila RN Lynfield R Comparison of community- and health care-associated methicillin-resistant *Staphylococcus aureus* infection JAMA 2003 290 22 2976 2984 10.1001/jama.290.22.2976 14665659

[B6] Dukic VM Lauderdale DS Wilder J Daum RS David MZ Epidemics of community-associated methicillin-resistant *Staphylococcus aureus* in the United States: a meta-analysis PLoS One 2013 8 1 e52722 10.1371/journal.pone.0052722 23300988 PMC3534721

[B7] Herold BC Immergluck LC Maranan MC Lauderdale DS Gaskin RE Boyle-Vavra S Leitch CD Daum RS Community-acquired methicillin-resistant *Staphylococcus aureus* in children with no identified predisposing risk JAMA 1998 279 8 593 598 10.1001/jama.279.8.593 9486753

[B8] David MZ Daum RS Community-associated methicillin-resistant *Staphylococcus aureus*: epidemiology and clinical consequences of an emerging epidemic Clin Microbiol Rev 2010 23 3 616 687 10.1128/CMR.00081-09 20610826 PMC2901661

[B9] Moran GJ Krishnadasan A Gorwitz RJ Fosheim GE McDougal LK Carey RB Talan DA Methicillin-resistant *S. aureus* infections among patients in the emergency department N Engl J Med 2006 355 7 666 674 10.1056/NEJMoa055356 16914702

[B10] Klevens RM Morrison MA Nadle J Petit S Gershman K Ray S Harrison LH Lynfield R Dumyati G Townes JM Craig AS Zell ER Fosheim GE McDougal LK Carey RB Fridkin SK Invasive methicillin-resistant *Staphylococcus aureus* infections in the United States JAMA 2007 298 15 1763 1771 10.1001/jama.298.15.1763 17940231

[B11] Chamchod F Ruan S Modeling the spread of Methicillin-Resistant *Staphylococcus aureus* in nursing homes for elderly PLoS One 2012 7 1 e29757 10.1371/journal.pone.0029757 22238650 PMC3253090

[B12] Meng Y Davies R Hardy K Hawkey P The application of agent-based simulation to the modelling of MRSA transmission in hospital J Simulation 2010 4 60 67 10.1057/jos.2009.17

[B13] Barnes S Golden B Wasil E MRSA transmission reduction using agent-based modeling and simulation INFORMS J Computing 2010 22 4 635 646 10.1287/ijoc.1100.0386

[B14] D’Agata E Webb G Horn M Moellering R Ruan S modeling the invasion of community-acquired methicillin-resistant *Staphylococcus aureus* into hospitals Clin Infect Dis 2009 48 3 274 284 10.1086/595844 19137654 PMC2666259

[B15] Forrester M Comm B Arts B Pettitt A Use of stochastic epidemic modeling to quantify transmission rates of colonization with Methicillin‒Resistant *Staphylococcus aureus* in an intensive care unit Infect Control Hosp Epidemiol 2005 26 7 598 606 10.1086/502588 16092739

[B16] Hotchkiss J Strike D Simonson D Broccard A Crooke P An agent-based and spatially explicit model of pathogen dissemination in the intensive care unit Crit Care Med 2005 33 1 168 176 10.1097/01.CCM.0000150658.05831.D2 15644665

[B17] Raboud J Saskin R Simor A Loeb M Green K Low D McGeer A Modeling transmission of Methicillin-Resistant *Staphylococcus aureus* among patients admitted to a hospital Infect Control Hosp Epidemiol 2005 26 7 607 615 10.1086/502589 16092740

[B18] Sebille V Valleron AJ A computer simulation model for the spread of nosocomial infections caused by multidrug-resistant pathogens Comput Biomed Res 1997 30 307 322 10.1006/cbmr.1997.1451 9339324

[B19] Lee B McGlone S Wong K Yilmaz L Avery T Song Y Christie R Eubank S Brown S Epstein JM Parker J Burke D Platt R Huang S Modeling the spread of Methicillin-Resistant *Staphylococcus aureus* (MRSA) outbreaks throughout the hospitals in Orange County, California Infect Control Hosp Epidemiol 2011 32 6 562 572 10.1086/660014 21558768 PMC3388111

[B20] Bootsma MC Diekmann O Bonten MJM Controlling methicillin-resistant *Staphylococcus aureus*: Quantifying the effects of interventions and rapid diagnostic testing Proc Natl Acad Sci 2006 103 5620 5625 10.1073/pnas.0510077103 16565219 PMC1459403

[B21] Ke W Huang SS Hudson LO Elkins KR Nguyen CC Spratt BG Murphy CR Avery TR Lipsitch M Patient sharing and population genetic structure of methicillin-resistant *Staphylococcus aureus* Proc Natl Acad Sci 2012 109 17 6763 6768 10.1073/pnas.1113578109 22431601 PMC3340079

[B22] Hogea C VAN Effelterre T Acosta CJ A basic dynamic transmission model of *Staphylococcus aureus* in the US population Epidemiol Infect 2013 23 1 11 10.1017/S0950268813001106 PMC3915753 23701989

[B23] Cooper BS Medley GF Stone SP Kibbler CC Cookson BD Roberts JA Duckworth G Lai R Ebrahim S Methicillin-resistant *Staphylococcus aureus* in hospitals and the community: Stealth dynamics and control catastrophes Proc Natl Acad Sci 2004 101 27 10223 10228 10.1073/pnas.0401324101 15220470 PMC454191

[B24] Wheaton WD Cajka JC Chasteen BM Wagener DK Cooley PC Ganapathi L Roberts DJ Allpress JL Synthesized population databases: a US geospatial database for agent-based models. Publication No. MR-0010-0905 2009 North Carolina: RTI Press 10.3768/rtipress.2009.mr.0010.0905 PMC2875687 20505787

[B25] Centers for Disease Control and Prevention (CDC) Methicillin-resistant Staphylococcus aureus infections in correctional facilities–Georgia, California, and Texas, 2001–2003 MMWR Morb Mortal Wkly Rep 2003 52 41 992 996 14561958

[B26] Pan ES Diep BA Carleton HA Charlebois ED Sensabaugh GF Haller BL Perdreau-Remington F Increasing prevalence of methicillin-resistant *Staphylococcus aureus* infection in California jails Clin Infect Dis 2003 37 10 1384 1388 Nov 15 10.1086/379019 14583874

[B27] David MZ Mennella C Mansour M Boyle-Vavra S Daum RS Predominance of methicillin-resistant *Staphylococcus aureus* among pathogens causing skin and soft tissue infections in a large urban jail: risk factors and recurrence rates. J Clin Microbiol 2008 46 10 3222 3227 10.1128/JCM.01423-08 18685002 PMC2566069

[B28] Feld S The focused organization of social ties Amer J Sociology 1981 86 5 1015 1035 10.1086/227352

[B29] Fritz SA Camins BC Eisenstein KA Fritz JM Epplin EK Burnham CA Dukes J Storch GA Effectiveness of measures to eradicate *Staphylococcus aureus* carriage in patients with community-associated skin and soft-tissue infections: a randomized trial Infect Control Hosp Epidemiol 2011 32 9 872 880 10.1086/661285 21828967 PMC3528015

[B30] Miller L Eells S Taylor A David M Ortiz N Zychowski D Kumar N Cruz D Boyle-Vavra S Daum R *Staphylococcus aureus* colonization among household contacts of patients with skin infections: risk factors, strain discordance, and complex ecology Clin Infect Dis 2012 54 11 1523 1535 10.1093/cid/cis213 22474221 PMC3348950

[B31] Larsson AK Gustafsson E Nilsson AC Odenholt I Ringberg HK Melander E Duration, of methicillin-resistant *Staphylococcus aureus* colonization after diagnosis: A four-year experience from southern Sweden Scand J Infect Dis 2011 43 6–7 456 462 doi: 10.3109/00365548.2011.562530. Epub 2011 Mar 2 21366406 10.3109/00365548.2011.562530

[B32] Ellis MW Hospenthal DR Dooley DP Gray PJ Murray CK Natural history of community - acquired methicillin - resistant *Staphylococcus aureus* colonization and infection in soldiers Clin Infect Dis 2004 39 7 971 979 10.1086/423965 15472848

[B33] Craig BA Sendi PP Estimation of the transition matrix of a discrete-time Markov chain Health Econ 2002 11 33 42 10.1002/hec.654 11788980

[B34] Gorwitz RJ Kruszon-Moran D McAllister SK McQuillan G McDougal LK Fosheim GE Jensen BJ Killgore G Tenover FC Kuehnert MJ Changes in the prevalence of nasal colonization with *Staphylococcus aureus* in the United States, 2001–2004 J Infect Dis 2008 197 9 1226 1234 10.1086/533494 18422434

[B35] Hota B Ellenbogen C Hayden MK Aroutcheva A Rice TW Weinstein RA Community-associated methicillin-resistant *Staphylococcus aureus* skin and soft tissue infections at a public hospital: Do public housing and incarceration amplify transmission? Arch Intern Med 2007 167 10 1026 1033 10.1001/archinte.167.10.1026 17533205

[B36] Sargent RG Verification and validation of simulation models J Simulation 2012 7 12 24

[B37] Epstein JM Generative Social Science: Studies in Agent-Based Computational Modeling 2007 Princeton: Princeton University Press

[B38] North MJ Collier NT Ozik J Tatara ER Macal CM Bragen M Sydelko P Complex adaptive systems modeling with Repast Simphony Complex Adapt Syst Model 2013 1 1 3 10.1186/2194-3206-1-3

[B39] Collier NT North MJ Parallel Agent-based Simulation with Repast for High Performance Computing, Simulation 2012 Online November 6, 2012, doi:10.1177/0037549712462620

[B40] Macal CM North MJ Collier NT Dukic VM Lauderdale DS David MZ Daum RS Shumm P Daum RS Evans JA Wilder JR Wegener DT Laroque C, Himmelspach J, Pasupathy R, Rose O, Uhrmacher AM Modeling the Spread of Community-Associated MRSA Proc. 2012 Winter Simulation Conf 2012 Available at http://www.informs-sim.org/wsc12papers/includes/files/inv214.pdf (accessed June 20, 2013)

