# Supplementary material for: Modeling the transmission of community-associated methicillin-resistant Staphylococcus aureus: a dynamic agent-based simulation
Source: J Transl Med. 2014 May 12;12:124. doi: 10.1186/1479-5876-12-124 (PMC4049803; doi:10.1186/1479-5876-12-124)
Supplement: Additional file 1 — Technical appendix: supplemental information. [file 1479-5876-12-124-S1.docx]

### TECHNICAL APPENDIX: SUPPLEMENTAL INFORMATION

**Overview of Processes in the CA-MRSA Agent-Based Model**

The logical flow of processes in the CA-MRSA Agent-Based Model (CA-MRSA ABM) is shown in Fig. S1. The simulation is initialized at hour zero with agents in CA-MRSA disease states located across Chicago. The Mobility/Activity Model advances agent locations appropriate for the hour of the day, moving agents between their own households, schools, workplaces, other households, etc. Specialized mobility/activity models are developed separately to model the circulation of agents from the community into and out of hospitals and into and out of the Cook County Jail, to represent specific locations that are potentially important for MRSA transmission but are infrequently or never visited by most agents. For each place in the community, if colonized or infected agents are present, a Contact Transmission Model is activated to determine whether uncolonized agents at that location become colonized. A Disease State Transition Model (described in the main paper) is also activated to determine whether colonized and infected agents at that location transition to other states. Once disease state changes are recorded, or logged, the hour is incremented and the simulation processes are repeated until the simulation reaches the end time, at which point yearly and hourly summary reports are produced.

**MRSA Risk Categories**

The likelihood of MRSA transmission depends in part on the amount of physical contact in the place/activity at which the contact occurs. Levels are designated as high, moderate or negligible. Place/activities are assigned one of three levels of risk. MRSA risk categories by place and activity are summarized in Table S1.

### Modeling Behavior

### Ferguson [1] contends that understanding the dynamics of infectious-disease transmission demands a holistic approach, and several recent simulation models of epidemics have incorporated endogenous behavior of agents who dynamically respond to disease states computed by the model. Del Valle et al. [2] used a simplified, compartmentalized model of an epidemic outbreak that included behavioral changes modeled by the permanent transfer of individuals during the outbreak to a less active class; transfer rates depended on people’s knowledge of the outbreak as measured by the number of identified cases. Epstein et al. [3] modeled two interacting compartmentalized contagion processes: one of disease and one of fear of the disease. Aleman et al. [4] modeled the actions of individuals based on the recognition of a pandemic outbreak; as people realized an outbreak was occurring, they decided to quarantine themselves, admit themselves to hospitals, and otherwise change behavior. Lizon et al. [5] incorporated patient behaviors and interactions with healthcare workers into their model. However, there has been little attention to the role of behavior in infectious disease spread when there is not a well-publicized epidemic.

### We have developed a general framework for modeling behavior of patients, and potentially of healthcare workers. Fig. S2 shows a scenario in which an individual agent acquires a CA-MRSA infection for the first time and decides whether to seek care or to self-care. The behavioral framework shown explicitly represents the infection duration, actions the agent might take in response to the infection, and a possible public health intervention. An alternative behavioral model in future versions of the model might be invoked for the same agent if infection reoccurs; for example, the next time the agent develops an infection, they might seek treatment more quickly and receive more intense treatments. To implement this behavior, an agent would “remember” the timing and outcome of a previous infection if we were to add a memory attribute to the agents in the model, which the model can readily accommodate. For example, if the person sought treatment the first time and the infection resolved quickly, that could lead the person to seek early treatment for the next infection. Similarly, if the person self-treated previously and the infection resolved, that could make the person decide to self-treat again for the next infection. However, if self-care for the initial infection goes badly, the initial infection is particularly severe, or the infection takes a long time to resolve, that might enhance the likelihood of seeking care for the later infection. Problems with an infection treated by a clinician might only enhance the likelihood of seeking treatment for a later infection.

**Model Experiments and Sensitivity Analysis.**

Fig. S3 compares simulated CA-MRSA infection incidence with the estimated number of infections for combinations of plausible values of three transmission/transitions parameters, which are parameters in the model having the greatest uncertainty: uncolonized to colonized rate of transition, colonized to uncolonized rate of transition; and colonized to infected rate of transition. Ranges for the three parameters were established from the baseline estimates of the parameters. Within the respective ranges, five values were established for each parameter consisting of -50%, -25%, 0% (baseline value), +25%, and +50% of the parameter baseline value. As can be seen in Fig. S3, some simulations overshoot the estimated infection incidence build-up (gray), and some runs underestimate the build-up (orange and black). A few runs result in incidence levels remaining at or below 2002 levels for the duration of the simulation (black). Some runs match the build-up within the range of assumed uncertainty (blue). These results suggest the degree of sensitivity of the eventual outcome of the CA-MRSA incidence to the disease transmission/transition rates and identify the cases in which the epidemic would not have occurred at all.

Fig. S4 shows the results of a parameter sweep over the space of transmission and transition parameters *a*, *b*, and *e*. We hypothesized that increasing or decreasing the colonization rate *(*parameter *a)* and the decolonization rate (parameter *e)* at the same time (possibly by different relative amounts) would also result in good fits to the empirical data. The results in Fig. S4 verified our hypothesis (a near linear scaling relationship between *a* and *e* across values of the infection rate (*b).* The blue cells along the diagonal illustrate the key and expected relationship between colonization and decolonization rates that produce good fits to the meta-data logistics curve across the range of infection rates.

**Stochastic Variability in the Simulation**

The CA-MRSA ABM is inherently a stochastic model. Stochasticity enters into the process of disease transmission (uncolonized to colonized transition) and to each transition (the length of time in the colonized or infected states). The stochastic nature of the simulation also requires many simulation runs to properly characterize uncertainty. Studies were performed to determine the appropriate batch size for the number of simulation runs in a batch to characterize the extent of uncertainty. We estimate the variance in the infection incidence as a function of the number of simulation runs considered. Fig. S5 plots the variance of the infection incidence as a function of the number of simulation runs in which the only variation is the random number seed to initialize the run. Fig. S5 shows that the moving average of the estimated variance decreases continuously from 15 to about 28 runs and then stabilizes, indicating that batch sizes of a minimum of 28 runs are adequate to estimate of model output statistics. We run simulations in which each batch consists of 32 runs.

**References**

1. Ferguson N (2007) Capturing human behavior. *Nature* 446: 733.
2. Del Valle S, Hethcote H, Hyman JM, Castillo-Chavez C (2005) Effects of behavioral changes in a smallpox attack model. *Mathematical Biosciences*. 195(2): 228-251.
3. Epstein J, Parker J, Cummings D, Hammond R (2008) Coupled contagion dynamics of fear and disease: mathematical and computational explorations. *PLoS ONE* 3(12): e3955.
4. Aleman D, Wibisono T, Schwartz B (2011) A non-homogeneous mixing model for predicting pandemic disease spread. *Interfaces*, Special Issue on Humanitarian Applications 41(3): 301-315.
5. Lizon N, Aleman D, Schwartz B (2010) Incorporating healthcare systems in pandemic models, *Proc. 2010 Winter Simulation Conf.*, Johansson B, Jain S, Montoya-Torres J, Hugan J, Yucesan E, eds. 2230-2236. Wiley-IEEE Press.

**FIGURES AND TABLES FOR TECHNICAL APPENDIX**

**Figure S1.** CA-MRSA agent-based model process logic.

**Figure S2**. Discrete event framework for modeling behavior-based disease state transitions.

**Figure S3**. Comparison of simulated CA-MRSA infection incidence with estimated number of infections for various assumptions for transmission and transitions parameters.

**Figure S4**. Results of parameter sweep over space of transmission and transition parameters a, b, and e.

**Figure S5**. Simulation stochasticity exhibited by 32 model runs (a), and stochastic variability as a function of batch size (b).

**Table S1**. Assumed MRSA risk for key places.

**Figure S1.** CA-MRSA agent-based model process logic.

### Figure S2. Discrete event framework for modeling behavior-based disease state transitions.

**Figure S3.** Comparison of simulated CA-MRSA infection incidence with estimated number of infections for various assumptions for transmission and transitions parameters.


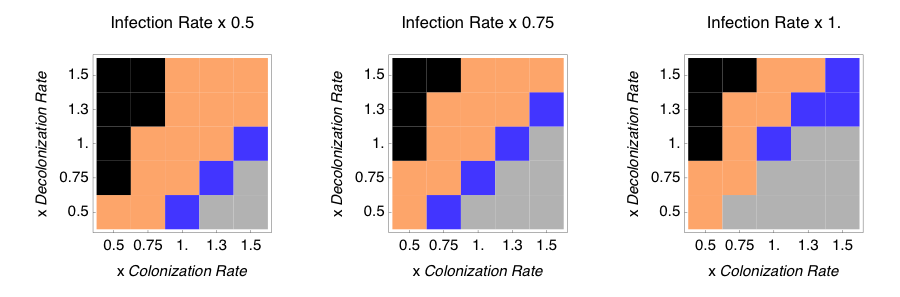


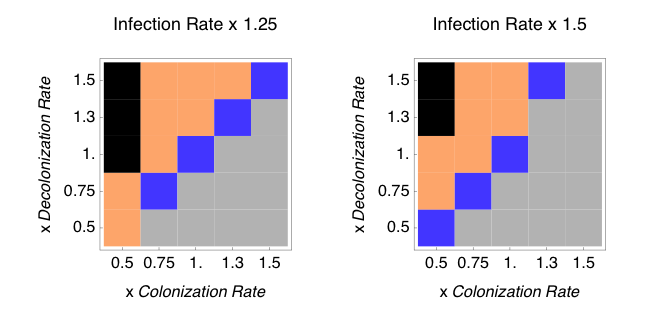


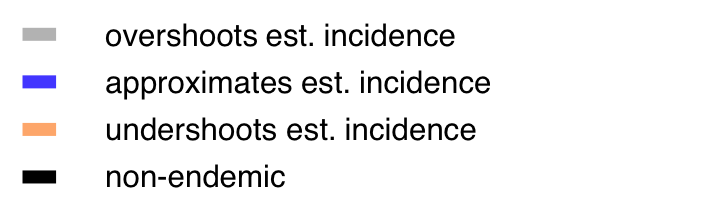


**Figure S4.** Results of parameter sweep over space of transmission and transition parameters *a*, *b*, and *e*. The blue cells along the diagonal illustrate the key relationship between colonization and decolonization rates that produce good fits to the meta-data logistics curve across the range of infection rates.

## a b

## Figure S5. Simulation stochasticity exhibited by 32 model runs (a), and stochastic variability as a function of batch size (b). The plot shows the variance of the infection incidence at the end of the simulation (year 2010) computed for batch sizes ranging from 3 to 32.

**Table S1.** Assumed MRSA risk for key places.

| **MRSA Risk Category** | **Number in Chicago** | **Place/Activity Risk (PAR)** | **Notes** |
| --- | --- | --- | --- |
| *High Risk:* |  |  |  |
| Households | 1,074,993 | 2 | Households located in Chicago proper |
| Hospitals | 95 | 2 | Hospital Circulation Model, operates on hospitals utilized by Chicago residents |
| Jails | 31 | 2 | Jail Circulation Model, focuses on the Cook County jail complex |
| Nursing homes | 272 | 2 | Considered as part of group quarters |
| Gyms | 309 | 2 | Considers gyms utilized by Chicago residents |
| *Moderate Risk:* |  |  |  |
| Schools/Daycare | 2,004 | 1 | Daycare and school children are segregated into classrooms by age |
| College dormitories | 36 | 1 | Considered as part of group quarters |
| *Negligible Risk:* |  |  |  |
| Workplaces | 116,716 | 0.1 | Located throughout Cook and DuPage Counties |

Note: Regardless of place, sports activities have higher transmission rates implemented by an Activity Intensity Parameter (AIP) = 2.
